# Instruction in Tropical Medicine

**Published:** 1905-09-02

**Authors:** 


### Instruction in Tropical Medicine.

The most important advance which has been made in recent years in the science of medicine has been the elucidation of the mysteries of certain very prevalent and destructive tropical diseases, and the discovery of means to prevent their spread or to abate their violence. This knowledge has already deprived large areas of country of their terrors for the white man, and as time goes by this influence promises to render habitable vast regions which in the past have been too unhealthy for Europeans. The economic value of these accessions of knowledge, and the wide possibilities which they open up have been recognised by the Government, and it is their intention to require in the future that medical officers in the public services shall undergo a special training in the diseases of the tropics and in the measures for their treatment and prevention.

It is now competent, for those who desire it, to obtain a University Diploma in Tropical Medicine, and this may be taken either at Cambridge or Liverpool. At Cambridge the fee is 6 guineas, and the examination is held in August. The Liverpool examinations are held three times a year, and the fee is 5 guineas. The University of Edinburgh, also, gives a special certificate in diseases of tropical climates and is now contemplating the institution of a diploma in this branch of medicine. In all instances, both teaching and examinations are open to women as well as to men. At Liverpool there is a short course given to qualified nurses; the fee is 1 guinea.

The London School of Tropical Medicine.

The London School is situated near the Royal Victoria and Albert Docks, E., and is connected with the Branch Hospital of the Seamen's Hospital Society. The position of the school and hospital ensures a constant supply of clinical material as the patients come from vessels trading with the tropics into the docks which adjoin the Hospital and School. Hence acute cases of many diseases peculiar to the tropics can always be studied, and adequate provision is made for clinical instruction by the hospital staff. The laboratory course occupies necessarily a prominent part of the curriculum and includes daily practical teaching in the use of the microscope, the examination of the blood, and the recognition of the various parasites which are known to play so prominent a part in the production of tropical diseases. The full curriculum extends over three months and the inclusive fee is 16 guineas. In each year there are three sessions, beginning on October 1st, January 15th, and May 1st, respectively. Provision is made for the accommodation of a certain number of resident students for whom, also, board is provided. This is a great advantage, as the curriculum is a very full one, and those who wish to pursue it thoroughly will be well advised to go into residence. Lastly, a word may be said of the help afforded by daily tutorial teaching given by a capable and experienced medical tutor. Applications for admission to the school should be made as early as possible, as the number of students desirous of coming into residence is greater than the School can accommodate.

Since the opening of the School 522 students have been in attendance including 37 women graduates. The teaching is so arranged as to provide a complete course of instruction for the Cambridge diploma in Tropical Medicine.

### The Liverpool School of Tropical Medicine.

The Liverpool School of Tropical Medicine conducts its laboratory work at University College and its courses of clinical instruction at the Royal Southern Hospital, where a special ward is allotted to the reception of cases of tropical disease. At so important a port there is, of course, always a large seafaring population, and thus clinical opportunities are well represented. The fee for a full course (lasting about two months) is 10 guineas. A hall of residence is attached to the school.

